# Supplementary material for: Uptake of diabetic retinopathy screening at a secondary level facility in Malawi
Source: PLOS Glob Public Health. 2023 Nov 8;3(11):e0002567. doi: 10.1371/journal.pgph.0002567 (PMC10631633; doi:10.1371/journal.pgph.0002567)
Supplement: S1 Checklist — (DOCX) [file pgph.0002567.s001.docx]

STROBE Statement—checklist of items that should be included in reports of observational studies

|  | Item No. | Recommendation | Page  No. | Relevant text from manuscript |
| --- | --- | --- | --- | --- |
| **Title and abstract** | 1 | (*a*) Indicate the study’s design with a commonly used term in the title or the abstract | 2 | Cross sectional study |
|  |  | (*b*) Provide in the abstract an informative and balanced summary of what was done and what was found | 2-3 | **What was done:** Consecutive patients were interviewed using a structured questionnaire to record their characteristics, medical details including visual acuity and data regarding; the frequency of clinic visits, knowledge of existence of DR services and a history of referral for DR screening in the prior one year. The outcome variable was uptake for DR screening over one year. Univariate binary logistic regression was used to investigate predictors of uptake and explanatory variables that had a P-value of < 0.1 were included into a multivariate logistic regression model.  **What was found:** We recruited 230 participants over a three-month period with a median age of 52.5 years (IQR 18-84) and a median duration of diabetes of 4 years (IQR 1-7). The average interval of clinic visits was 1.2 months (SD ± 0.43) and only 59.1% (n = 139) of the participants were aware of the existence of diabetic retinopathy screening services at the facility. The uptake for DR screening over one year was 20% (n = 46). The strongest predictors of uptake were awareness of the existence of DR screening services (OR 10.05, P <0.001) and a history of being referred for DR screening (OR 9.02, P <0.001) and these remained significant on multivariable analysis. |
| Introduction | | | |  |
| Background/rationale | 2 | Explain the scientific background and rationale for the investigation being reported |  | Most countries in Sub Saharan Africa lack the resources required to set up and maintain systematic DR screening programs [18] . Thus opportunistic screening for DR is often utilized in most screening programs in the region. This involves providing DR screening to patients with diabetes upon their presentation to a health facility to seek DM care [18]. Considering the shortage of health facilities, personnel skilled in DR screening and the poor accessibility to facilities offering this service, diabetic retinopathy may go unnoticed in most patients. Thus most patients present to eye health facilities only after experiencing considerable vision loss [2]. It is thus important to establish interventions that may promote uptake of DR screening in an opportunistic DR screening program. (Line 83 to 91) |
| Objectives | 3 | State specific objectives, including any prespecified hypotheses |  | We studied the uptake and factors associated with uptake of DR screening in an opportunistic DR screening program at a secondary level diabetes clinic in Southern Malawi (103 to 105) |
| Methods | | | |  |
| Study design | 4 | Present key elements of study design early in the paper | 6 | This cross-sectional study was carried out over 3 months in the diabetes clinic (108) |
| Setting | 5 | Describe the setting, locations, and relevant dates, including periods of recruitment, exposure, follow-up, and data collection | 6-7 | The study was carried out over 3 months in the diabetes clinic at Thyolo district hospital between the months of October 2020 to December 2020. The hospital is a secondary level health facility in Southern Malawi and the population of its catchment area is about 721,456 people. At the time of the study, the diabetes clinic at the facility had a total of 700 patients on its register of patients. The study population consisted of all patients attending the diabetes clinic at the hospital over the study period. As part of routine care, all patients attending the clinic are informed of the need for having an eye examination at least once a year to screen for ocular complications of diabetes. The eye examinations are performed in a room that is adjacent to the diabetes clinic. Our study included all consecutive patients aged at least 18 years old who presented to the diabetes clinic. Written informed consent was obtained from all participants before data collection interviews and the only criterion for exclusion was a refusal to participate in the study. All data was deidentified upon transcription onto the data collection sheet. (108-121) |
| Participants | 6 | (*a*) *Cohort study*—Give the eligibility criteria, and the sources and methods of selection of participants. Describe methods of follow-up  *Case-control study*—Give the eligibility criteria, and the sources and methods of case ascertainment and control selection. Give the rationale for the choice of cases and controls  *Cross-sectional study*—Give the eligibility criteria, and the sources and methods of selection of participants | 7 | Our study included all consecutive patients aged at least 18 years old who presented to the diabetes clinic. Written informed consent was obtained from all participants before data collection interviews and the only criterion for exclusion was a refusal to participate in the study. (line 117 to 120) |
|  |  | (*b*) *Cohort study*—For matched studies, give matching criteria and number of exposed and unexposed  *Case-control study*—For matched studies, give matching criteria and the number of controls per case |  |  |
| Variables | 7 | Clearly define all outcomes, exposures, predictors, potential confounders, and effect modifiers. Give diagnostic criteria, if applicable | 8 | The main outcome variable was the uptake of DR screening (which was defined as the proportion of patients who had a diabetic eye examination over the past one year). (line 137 to 139)  Continuous variables were summarized as means or medians at 95% confidence intervals and univariate logistic regression was used to investigate the association between uptake of DR screening and the following explanatory variables; age, sex, level of education, type of diabetes, duration of diabetes, awareness about DR screening services, history of referral for DR screening, intervals of clinic visits, history of hypertension, HIV status and the presenting visual acuity. Explanatory variables that had a p-value of < 0.1 on univariate analysis were included into a multivariate logistic regression model (line 140 to 147) |
| Data sources/ measurement | 8* | For each variable of interest, give sources of data and details of methods of assessment (measurement). Describe comparability of assessment methods if there is more than one group | 7 | A nurse interviewed each participant and ascertained the following data from the history and clinical records of each subject included in the study: age, sex, level of education, history of hypertension, type of diabetes, duration of diabetes, HIV status and the intervals at which the patient visited the clinic over the past 12 months. The nurse also asked each participant if they were aware of the existence of DR screening services at the facility, whether they had been referred to the OCO for DR screening within the prior 12 month period and whether they attended DR screening over the prior 12 months. After the interview, the visual acuity of each study participant was measured by an OCO using a Snellen chart. (Line 126 to 134) |
| Bias | 9 | Describe any efforts to address potential sources of bias | 7 | Our study included all consecutive patients aged at least 18 years old who presented to the diabetes clinic (Line 117 to 118) |
| Study size | 10 | Explain how the study size was arrived at |  |  |

Continued on next page

| Quantitative variables | 11 | Explain how quantitative variables were handled in the analyses. If applicable, describe which groupings were chosen and why | 8 | Continuous variables were summarized as means or medians at 95% confidence intervals and univariate logistic regression was used to investigate the association between uptake of DR screening and the following explanatory variables; age, sex, level of education, type of diabetes, duration of diabetes, awareness about DR screening services, history of referral for DR screening, intervals of clinic visits, history of hypertension, HIV status and the presenting visual acuity. Explanatory variables that had a p-value of < 0.1 on univariate analysis were included into a multivariate logistic regression model (line 140 to 147) |
| --- | --- | --- | --- | --- |
| Statistical methods | 12 | (*a*) Describe all statistical methods, including those used to control for confounding | 11 | Multivariate logistic regression |
|  |  | (*b*) Describe any methods used to examine subgroups and interactions | 11 | Multivariate logistic regression |
|  |  | (*c*) Explain how missing data were addressed |  | NA |
|  |  | (*d*) *Cohort study*—If applicable, explain how loss to follow-up was addressed  *Case-control study*—If applicable, explain how matching of cases and controls was addressed  *Cross-sectional study*—If applicable, describe analytical methods taking account of sampling strategy |  | NA |
|  |  | (*e*) Describe any sensitivity analyses |  | NA |
| Results | | | | |
| Participants | 13* | (a) Report numbers of individuals at each stage of study—eg numbers potentially eligible, examined for eligibility, confirmed eligible, included in the study, completing follow-up, and analysed | 8 | We recruited 230 participants over a three-month period and all eligible participants consented to participate in the study. (line 150 to 151) |
|  |  | (b) Give reasons for non-participation at each stage |  | NA |
|  |  | (c) Consider use of a flow diagram |  | NA |
| Descriptive data | 14* | (a) Give characteristics of study participants (eg demographic, clinical, social) and information on exposures and potential confounders | 10 | Table 2 (line 173) |
|  |  | (b) Indicate number of participants with missing data for each variable of interest |  | 0 |
|  |  | (c) *Cohort study*—Summarise follow-up time (eg, average and total amount) |  | NA |
| Outcome data | 15* | *Cohort study*—Report numbers of outcome events or summary measures over time |  | *NA* |
|  |  | *Case-control study—*Report numbers in each exposure category, or summary measures of exposure |  | *NA* |
|  |  | *Cross-sectional study—*Report numbers of outcome events or summary measures | *9* | screening uptake was 20% (line 159) |
| Main results | 16 | (*a*) Give unadjusted estimates and, if applicable, confounder-adjusted estimates and their precision (eg, 95% confidence interval). Make clear which confounders were adjusted for and why they were included | 9, 11 | Unadjusted estimates: The strongest predictors of uptake of DR screening over one year were being aware of the existence of DR screening services (OR 10.05, P <0.001) and a history of being referred for DR screening within the year (OR 9.02, P <0.001). Participants who had a diagnosis of hypertension had twice the odds (OR 2.13, P = 0.039) of having undergone of having undergone DR screening in the prior 12 months compared to those who did not have hypertension. (line 162 to 166)  Confounder-adjusted estimates: Explanatory variables that had a p-value of < 0.1 on univariate analysis were included into a multivariate logistic regression model (line 145 to 147) as shown in table 3 (line 180) |
|  |  | (*b*) Report category boundaries when continuous variables were categorized |  | NA |
|  |  | (*c*) If relevant, consider translating estimates of relative risk into absolute risk for a meaningful time period |  | NA |

Continued on next page

| Other analyses | 17 | Report other analyses done—eg analyses of subgroups and interactions, and sensitivity analyses |  | NA |
| --- | --- | --- | --- | --- |
| Discussion | | | | |
| Key results | 18 | Summarise key results with reference to study objectives | 3 | The uptake for DR screening over one year was 20% (n = 46). The strongest predictors of uptake were awareness of the existence of DR screening services (OR 10.05, P <0.001) and a history of being referred for DR screening (OR 9.02, P <0.001) and these remained significant on multivariable analysis. (line 37 to 40) |
| Limitations | 19 | Discuss limitations of the study, taking into account sources of potential bias or imprecision. Discuss both direction and magnitude of any potential bias | 14 | A limitation of our study is that it did not have a qualitative component looking into the consumer-related reasons for attendance or non-attendance to DR screening especially among the patients who were aware of the existence of DR services (Line 238 to 240) |
| Interpretation | 20 | Give a cautious overall interpretation of results considering objectives, limitations, multiplicity of analyses, results from similar studies, and other relevant evidence | 14 | Our study showed that there was a low uptake for annual eye examinations through opportunistic DR screening at a secondary level diabetes clinic. Although awareness of DR screening services and referral for screening were strong predictors of uptake, there was low awareness of the existence of DR screening services at the facility and most patients were not individually referred for screening. (line 144 to 148) |
| Generalisability | 21 | Discuss the generalisability (external validity) of the study results | 14 | Our findings add to the limited body of literature on the uptake of DR screening in low income countries. Our findings are of value to policy makers and program managers of diabetic retinopathy screening programs in similar settings. (line 240 to 243) |
| Other information | |  | | |
| Funding | 22 | Give the source of funding and the role of the funders for the present study and, if applicable, for the original study on which the present article is based |  | The study project was funded by Malawi NCD BRITE consortium as a small mentored research grant. The funders had no role in the design of the study, data collection and interpretation of the results. The funding was for field work and participants compensantion fee. |

*Give information separately for cases and controls in case-control studies and, if applicable, for exposed and unexposed groups in cohort and cross-sectional studies.

**Note:** An Explanation and Elaboration article discusses each checklist item and gives methodological background and published examples of transparent reporting. The STROBE checklist is best used in conjunction with this article (freely available on the Web sites of PLoS Medicine at http://www.plosmedicine.org/, Annals of Internal Medicine at http://www.annals.org/, and Epidemiology at http://www.epidem.com/). Information on the STROBE Initiative is available at www.strobe-statement.org.
