# Supplementary material for: Uptake of diabetic retinopathy screening at a secondary level facility in Malawi
Source: PLOS Glob Public Health. 2023 Nov 8;3(11):e0002567. doi: 10.1371/journal.pgph.0002567 (PMC10631633; doi:10.1371/journal.pgph.0002567)
Supplement: Attachment — Submitted filename: Response to reviewers.docx [file pgph.0002567.s003.docx]

PLOS GLOBAL PUBLIC HEALTH

2 July 2023

Dear Editor,

Thank you for the reviewers’ feedback on the manuscript titled “**Uptake of diabetic retinopathy screening at a secondary level facility in Malawi”** by Thokozani Zungu, Shaffi Mdala, Petros Kayange, Elizabeth Fernado, Halima Twabi, Arnold Jumbe, Johnstone Kumwenda, Adamson Muula.

Below are the responses to each of the points that were raised.

A. **Editor’s comments**

**Introduction**

1. Lines 67-73: please explain the reason for including a description of UK DR screening programme? Is it relevant to your context?

**Response:** We had made reference to the UK DR screening programme to acknowledge it as the only DR screening programme that has been reported to achieve a high level of screening uptake. However, we acknowledge the lack of similarity of the UK programme to the Malawian context. We have thus removed the description of the UK programme in order to keep the introduction more focused on the Sub-Saharan African context and the Malawian context.

2. Lines 71-73: Why describe guidelines for a high-resource setting? Is it applicable to Malawi?

**Response:** We have reduced the description of the DR screening guidelines that are recommended for high-income settings. We have however acknowledged systematic DR screening as a method that has been proven to increase DR screening coverage in high income countries. This is with the purpose of highlighting the rationale why opportunistic screening is utilised in most low-income settings and the need for investigating the uptake achieved in this model of screening. (Line 67 to 73)

3. This section is too long; please shorten. The focus should be on Malawi with a clear motivation for conducting this study.

**Response:** We have removed some details about DR screening in high-income settings and added more context about the setup of DR screening services in Malawi. We have also highlighted the fact that there was no Malawian study prior to our study reporting on the uptake of DR screening. (Line 87 to 95)

4. Lines 74-82: This may be incorporated into the Discussion section where it can be compared with the findings in this study. Describing screening in Tanzania and Kenya is not a motivation for this study conducted in Malawi.

**Response:** This paragraph (Line 74-82) has been moved to the opening part of the discussion to highlight how the findings in Malawi compare with reports from the region. (Now line 217 to 224)

5. Line 81: please improve your grammar – “blinding eye complications”.

**Response:** The sentence has been rephrased and “blinding eye complication” has been replaced with “…..ocular complications” (Now line 247 to 248). The introduction was revised with this line rephrased and moved to the discussion section.

6. Lines 103-105: The aims should be clearly articulated. E.g., “The aim of this study was to…”

**Response:** We have rephrased the beginning of lines 103 -105 according to this advice (Now line 95 to 97)

**Methods**

7. Do patients pay for healthcare?
   Does it cost more for DR screening?
   Are the waiting times increased if patients undergo DR screening?
   Do patients self-select for DR screening?
   Are they not automatically provided with an appointment for DR screening?

**Response:** We have added a section on “study setting” to give clarity on the organisation of healthcare delivery and DR screening at the facility (Now line 99 to 124). In this regard, we have also removed information about the study setting from the “subjects” section (Line 108 to 117).

In summary, we have clarified that healthcare (including DR screening) is provided at no cost to patients and that patients are not automatically given an appointment. Rather, a clinic nurse provides health education to the patients as a group on the need for DR screening and at what intervals they should go for screening. Patients are expected to attend screening with respect to this advice. We have also stated that waiting time is increased when patients undergo DR screening. In the discussion section, one of our recommendations is research into the impact of interventions aimed at reducing patient waiting times on increasing screening uptake at the facility (such as introducing a dedicated DR screening clinic instead of the current set up where patients are seen in the general eye clinic for DR screening along with patients presenting due to other ophthalmic conditions).

8. Statistical analyses: what p-value level was considered statistically significant?

**Response:** Line 173: We have specified that variables that had a p-value of <0.05 were considered to be statistically significant.

**Results**

9. Were only 230 patients seen at the clinic over a 3-month period - October 2020 to December 2020?

**Response:** More than 230 patients were seen over the three months period.

We have clarified on the study setting, (Line 101 to 103) that the clinic is run on one day per week and about 30 patients with diabetes are seen in the clinic weekly. We recruited 230 consecutive patients as patients were recruited only once in the study. However, during month 2 and 3, some of the patients were follow ups that were presenting for review and these were not recruited again to avoid duplication.

10. Table 1: What does primary, secondary, etc. schooling refer to? This translates to how many years of schooling completed?

**Response:** Primary education takes eight years and secondary education is four years long. In this study we analysed level of education as a categorical variable regardless of the number of years or highest grade achieved at that level of education. We have thus clarified this by changing the terminology to level of education attended. (Line 170 and line 192 in table 1)

11. Use the word hypertension, not hypertensive.

**Response:** The table has been edited accordingly. (Line 192 table 1)

12. Please include HbA1c levels, if available.

**Response:** HbA1c levels are not routinely checked due to unavailability of resources at the facility. This has been acknowledged as a limitation in the final paragraph of the discussion (Line 324 to 326).

13. Table 1 and 3: please write 1.00 for the reference value for the logistic regression.
    Note, this should be presented first.

**Response:** 1.00 has been written as the reference value for the logistic regression and the reference variables have been presented first. Table 1 and Table 2 (Line 192 to line 193 and line 213 to line 214).

14. Where is Table 2?

**Response:** The caption “Table 3” was an error and we have corrected this to “Table 2” (Line 213 to 214). There are only two tables in the results section.

**Discussion**

15. Line 186: name the countries and describe the comparable, or otherwise, prevalence.

**Response:** This was moved from the introduction section and placed in the discussion section. It reads as… “In Tanzania, the uptake for DR screening at a tertiary eye hospital in the past 12 months was found to be 29% whilst in Kenya it was reported to be 13% (Line 222 to 224).

16. Lines 189-192: Please rephrase; there are numerous barriers, not a single one, for the absence of screening programmes.
    **Response:** Line 242 to line 249: This part of the discussion was rephrased to incorporate a more comprehensive list of barriers to accessing DR screening as reported from the literature.
17. Lines 203-204: What does “…health promotion on the need for DR screening” actually entail? Please provide more detail.

**Response:** We have removed the word “health promotion” to improve clarity. We have rephrased the point by stating that the patients routinely received health education on the need for DR screening during each clinic visit” (Line 258 to 259). In addition, under the study setting section, we have clarified that health education is part of routine care at the clinic (Line 114 to 120).

18. Why are all patients not routinely referred for DR screening at this diabetes clinic? How are patients selected for DR screening?

**Response:** This has been clarified in the study setting section (Line 114 to line 120) as follows:

“As part of routine care, a group-based health education session is delivered by a nurse during each clinic and it includes information for patients on the need for annual DR screening. The nurse-led diabetes health education is meant to reduce the amount of time that the clinician running the DM clinic spends on counselling each patient about the complications of DM and the need for DR screening. DR screening is performed in the eye clinic at the facility and patients attend screening according to the schedule advised to them during the group health education sessions.”

In the discussion section (line 269 to 276), we have pointed out gaps with this screening approach and made a recommendation that can improve the referrals.

19. Line 220: What do you mean by ‘health care demand’?

**Response:** We intended to put across the point that there is evidence that comorbidity increases health care utilization (i.e. the extent to which individuals make use of health care services to meet their medical needs. It is also a mesure of the frequency at which healthcare services are utilised by an individual within a given time period). “Health care demand” was a less appropriate term for this and we have thus dropped it. (Line 285 to line 287)

20. Line 222: Uptake of what?

**Response:** The line has been revised to read as “uptake of DR screening” (Now line 290). With respect to this advice we have revised the whole manuscript on all points where there was a need to specify on the meaning of uptake.

21. Line 224: replace ‘weak’ with non-significant’. What was the reason for this? Likely the small sample size. Please include.

**Response:** This has been amended by replacing “weak” with “non-significant” and acknowledging small sample size as a possible reason. (line 288 to 292)

22. Lines 232-233: Again, the small sample size with only 49 patients screened is likely the reason for the lack of significance and should be stated.

**Response:** Small sample size has been acknowledged as a possible reason for the non-signficance (line 312 to 316)

23. Line 235: please explain: ’among different races’ – why is race relevant to this study?

**Response:** Race was not relevant to this study and the statement has been removed from the manuscript to improve clarity.

24. **Recommendations:** Please provide clear recommendations to improve DR at this clinic; these should be linked to your data. What are areas for further research?

**Response:** Recommendations to improve DR screening at this clinic have been added on the following lines:

- Line 234 to line 239: We thus recommend research into the effectiveness of interventions aimed at reducing patient waiting time on increasing uptake of DR screening. A possible intervention is the introduction of a dedicated DR screening clinic which would eliminate the time that patients wait on a queue of patients that present with other ophthalmic conditions in the general eye clinic.
- Line 261 to line 263: This warrants research into the feasibility and effectiveness of alternative health education models such as personalized counselling in DR screening and DM clinics in improving patients’ awareness about DR
- Line 268 to line 269: …. Thus, we recommend research into the effectiveness of peer-led health education to increase uptake of DR screening.
- Line 282 to line 284: We recommend that interventions to improve uptake of DR screening should promote referral by physicians for patients to undergo DR screening.

Areas of future research have been highlighted (Line 338 to line 341) as follows: “Future research should investigate interventions for promoting the referral by clinicians of patients with DM for DR screening. We also recommend research into the effectiveness of alternative health education models such as personalized health education and peer-led health education in improving uptake for DR screening.

25. Include the small sample size as a major limitation.

**Response:** The small sample size has been acknowledged as a limitation. Line 312 to line 314: …….the association between the variables is subject to being significantly influenced by the small number of participants that underwent DR screening.

26. Are there other such studies from Malawi or is it a first? Please include as a study strength.

**Response:** Line 327 to line 331: One strength of this study is that it is the first study to report on the uptake of DR screening at secondary level in Malawi and the findings add to the limited body of literature…..

27. You did not examine glycaemic control as a factor for DR; this is a limitation. If this data are available, please include in your analyses.

**Response:** Line 323 to 326: We have acknowledged that “due to lack of facilities for performing HbA1C at the study location, we could not investigate the relationship between uptake of DR screening and patients’ glycemic control.”

28. Line 249-254: Please move recommendations and future research to the Discussion section and link it directly to a study finding.

**Response:** Recommendations to improve DR screening at this clinic have be stated and linked to study findings as follows:

Line 234 to line 239: We thus recommend research into the effectiveness of interventions aimed at reducing patient waiting time on increasing uptake of DR screening. A possible intervention is the introduction of a dedicated DR screening clinic which would eliminate the time that patients wait on a queue of patients that present with other ophthalmic conditions in the general eye clinic.

Line 261 to line 263: This warrants research into the feasibility and effectiveness of alternative health education models such as personalized counselling in DR screening and DM clinics in improving patients’ awareness about DR

Line 268 to line 269: …. Thus, we recommend research into the effectiveness of peer-led health education to increase uptake of DR screening.

Line 282 to line 284: We recommend that interventions to improve uptake of DR screening should promote referral by physicians for patients to undergo DR screening.

B. **Reviewers’ Comments**

**Reviewer #1:**

The article addressed a relevant issue that requires more research, especially in low and middle-income countries.
However, the text lacks key contextual information to allow the readers to understand the many barriers people with diabetes face to access timely health care in Malawi.
For example, the article states that the prevalence of DM adults is 2% in rural areas and 3% in urban areas. However, the information comes from a cross-sectional study that, because of the sample size, provides only an estimate, shows that DM is a problem in the country and that there is a high prevalence of risk factors for developing diabetes.
There needs to be more information about Malawi’s health system:

1. how many people have access to health insurance, out-of-pocket expenses, if the DR screening program offers the exam free of cost

**Response:** This has been explained in the study setting paragraph as follows: ……All healthcare services in public hospitals in Malawi, including DR screening, are government-funded and free to all patients at the point of delivery. (Line 104 to 107)

2. if the country has a DM program and policies. Also, there needs to be more information on the number of clinics that offer the DR screening programs, if these are located in urban or rural areas if there are regions or districts without facilities offering DR screening.

**Response:** We have described the study context as follows in the introduction section (Line 82 to 93)

The prevalence of DM among adults is 2% in rural areas and 3% in urban areas and the Ministry of Health has a Non-communicable diseases policy which includes DM management [17]. In the country’s national DR screening program, ophthalmic clinical officers (OCOs) and optometrists perform opportunistic DR screening using direct ophthalmoscopy with pharmacological pupillary dilatation. This is done among patients attending diabetes clinics in the country’s twenty-four district hospitals, which are all secondary level facilities that serve largely rural populations. The same cadre of eye health personnel performs DR screening among patients attending the country’s four tertiary-level diabetes clinics using slit lamps or fundus cameras. Patients identified with treatable retinopathy are referred for treatment to the tertiary level hospitals, which are located in the country’s four cities. There are no resources for DR screening in primary level health facilities.

3. The methods part has to be reviewed and enriched. Authors stated that the protocol adhered to the tenets of the Declaration of Helsinki for research involving human participants, and ethical approval was obtained from the College of Medicine Research and Ethics Committee (study protocol number P.10/19/2803), however, there is no information about the procedure. Informed consent is a process that must respect the autonomy of the participants, and as for this research, recruitment happened within the clinics and has 0 refusal rate; there is a need to know if: recruiters were workers at the clinics, how power relations were addresses, how autonomy was guarantee (beyond the informed consent if for example people were informed about the research in the day of their visit to the clinic, and where invited to come back another day for be part of the research).

**Response:** This has been clarified under study subjects in the methodology section Line 125 to 146: ……..The study included all consecutive patients aged at least 18 years old who attended the diabetes clinic at the hospital over the study period. A participant information sheet was used to explain about the study’s purpose and its procedures during the health education talks that the patients attend in a group before being seen in the clinic. Thereafter, the patients were individually invited to a private room where they were given a chance to ask questions about the study, written informed consent was obtained and data collection interviews were held. The collected data was de-identified upon transcription onto the data collection sheet. All explanations about the study and the informed consent process were done by a study nurse from Queen Elizabeth Central Hospital, which is the referral facility for Thyolo District Hospital.

The majority of the patients had a relative who had escorted them to the hospital and these signed as witnesses to the informed consent. Patients who had no witnesses were given an appointment to take part in the study on the following week after returning with a witness. Participants or witnesses who were illiterate signed the consent forms with a thumb print and the only criterion for exclusion was a refusal to participate in the study….

4. There needs to be more information about who covered the screening costs. This information is critical to describe how the ethics of the research was guaranteed.

**Response:** Line 147 to line 151: The study was funded by the Non-communicable Diseases Building Research Capacity, Implementation and Translation Expertise (NCD BRITE) consortium. The funding covered participant compensation fees, communication costs and transportation costs for the study team. The funders did not have any role in the study design and its implementation.

**Reviewer #2**

The article highlighted the importance of recognizing the need for referrals and management for diabetic retinopathy management in underresourced settings.

1. I would also comment if possible on the potential for scalability of this program in-country and methods for scalability and collaboration.

Response: We have commented on the potential for scalability of DR screening program in Malawi and methods of scalability and collaboration in the discussion section Line 296 to line 309 as follows…… “Similar to other low income countries, our study has highlighted lack of awareness and poor referral system for DR screening as potential areas to address for scalability of DR screening program in Malawi. In addition, the country’s health care also faces the challenges of poor infrastructure, inadequate human resource, poor data management and health information systems and limited treatment facilities [28]. With this background to the country’s general health care challenges that trickle down to its various health care programmes including the DR screening programme, the solution to these challenges demands a multi-disciplinary approach. There is need of raising awareness about DR and the importance of screening, improving the referral pathways and treatment facilities, investments in healthcare infrastructure, training and deploying more eye care workers and improving data management and health information systems. The success of scalability of DR screening program in Malawi would require commitment from local government in collaboration with international stakeholders in eye health and non-governmental organizations”..

It may also be helpful if doing further work after this study to collect information on HgA1c/glucose levels of patients that were referred or need referral for diabetic retinopathy screening.

**Response:** We have acknowledged lack of facilities for HbA1c at the study location as a limitation of the study. (Line 323 to line 326)

2. If possible, diabetic teaching and management could also be included during diabetic retinopathy screening as well.

**Response:** We have included this as a recommendation for the study

Line 261 to line 263: This warrants research into the feasibility and effectiveness of alternative health education models such as personalized counselling in DR screening and DM clinics in improving patients’ awareness about DR…….

**Reviewer #3**

Zungu T et al explore a key area in diabetes care of uptake of screening for diabetic retinopathy in a sub-Saharan African country.
Generally, the manuscript is well written with the rationale of the study well explained and the study methods used in data collection plus the exclusion and inclusion criteria well stated.
I have minor comments which the authors should respond to.

1. A more detailed description of the study site is needed. Does it serve a predominantly rural or urban area? How does have an effect on the observed low uptake of the screening services? Kindly elaborate.

**Response:** We have added a section on study setting (Line 99 to line 124). The study site serves predominantly a rural population.

We have elaborated further in Line 225 to line 230 that “the rural setting of the study location is a possible reason for the low uptake of DR screening in our study. We could not test this because there was no urban-based comparison group among the study population”

2. For the section on predictors of screening services on page 9 and in Table 1 on page 10, add 95% CI to aid in better interpretation of the results.

**Response:** 95% CI has been added on now page 12 (Line 197 to line 207) and in Table 1 on page 11 to page 12 (Line 192 to line 193).

3. Restrict your p-values to two decimal points apart from the highly significant ones of <0.001.

**Response:** This has been done in Table 1 and Table 2 and the rest of the manuscript

4. Could the authors also add a note on the availability and cost of treatment options of diabetes retinopathy in that hospital or in Malawi generally (both in the public and private sector) once a patient is diagnosed with severe forms of retinopathy like proliferative diabetic retinopathy.

**Response:** This has been elaborated on in the study setting section, Line 104 to line 107: All healthcare services in public hospitals in Malawi, including DR screening and treatment, are government-funded and are provided at no cost to all patients at the point of delivery…..

5. On page 6, the authors highlighted how diabetic screening is done at the different healthcare levels. I would like to know if this screening also includes assessment of other forms of ocular conditions like glaucoma which is also an identifiable cause of blindness in patients with diabetes. If so, how is it done? If it is not done, explain why.

**Response:** This has been explained further in the study setting section, Line 107 to line 112: ……Besides ophthalmoscopy, DR screening incorporates include visual acuity checks and anterior segment examination with a slit lamp to screen for other visually significant eye diseases that may affect patients with diabetes such as glaucoma, cataract and refractive error. In contrast to tertiary hospitals, there are no facilities for checking intraocular pressure or for performing ancillary tests such as perimetry in all district hospitals……
