# Supplementary material for: Uptake of diabetic retinopathy screening at a secondary level facility in Malawi
Source: PLOS Glob Public Health. 2023 Nov 8;3(11):e0002567. doi: 10.1371/journal.pgph.0002567 (PMC10631633; doi:10.1371/journal.pgph.0002567)
Supplement: Attachment — Submitted filename: Response to reviewers.odt [file pgph.0002567.s004.odt]

PLOS GLOBAL PUBLIC HEALTH
September 2023

Dear Editor,
Thank you for the reviewers’ feedback on the manuscript titled “Uptake of diabetic retinopathy screening at a secondary level facility in Malawi” by Thokozani Zungu, Shaffi Mdala, Petros Kayange, Elizabeth Fernado, Halima Twabi, Arnold Jumbe, Johnstone Kumwenda, Adamson Muula.
Below are the responses to each of the points that were raised.
Introduction
Lines 99-116: Please shift most of this text on the Malawian context to the Methods section.
Response: In line with the advice, Line 99 to 116 are in the methods section.
Results
The Editor disagrees with the Reviewer on the number of decimal places for p-values when reporting data. Please report all p-values to 3 decimal places in both tables and the main text, as is standard practice.
Response: All p-values have been revised to 3 decimal places in both tables and main text, Line 195 to line 196, line 208, line 214, line 216 to line 217
Discussion
Please improve the syntax of this section. There are many minor grammatical errors that need correction. I suggest a co-author (or anyone else) with an excellent grasp of the English language review this paper in detail for sentence construction, etc.
Response: We have done a major revision of the discussion section to address grammatical errors and improve sentence construction. 

Lines 290-294: This is an overly long poorly constructed sentence extending over 5 lines with ‘findings’ used twice in the sentence. Please rework.
Response: The section is in two sentences.( Line 217 to line 221)

Lines 294-296: Are the findings from the literature comparable to this study or not? Please comment; that is the purpose of a Discussion.
Response: The section has been revised for comparability of findings and reads as follows: . Long duration of DM was reported to be a strong predictor of uptake of DR screening in Kenya, India and among non-indigenous populations in Australia [13,29,30].  However, comparable to findings from Tanzania, long duration of DM was not an independent predictor of DR screening uptake in our study [20]. One possible explanation for this is the shorter median duration of DM of 4 years among our patient population. (with no track changes Line 287 to line 292)

Lines 297-305: How relevant is the urban-rural comparison to Malawi since most people (84%) live in rural areas? Please edit/rework the text.
Response: We have edited the paragraph by removing the line that read; “We could not test this because there was no urban-based comparison group among the study population”. (Paragraph 2 of discussion)
Lines 310-314: Please improve the clarity of these sentences as well as the syntax.
Response: The sentence construction of the whole discussion section has been revised.

Lines 330 and 441: Please replace ‘consumer’ with ‘patient’.
Response: The word “consumer “ has been replaced with patient (line 269 and line 311) 

Areas for future research should be ‘suggested’ and not ‘recommended’; please reword accordingly (used in multiple texts). The authors should be more circumspect.
Response: The word recommend has been replaced with suggest on sections discussing future research (line 242 and line 265) 

Line 370: replace ‘enough’ with ‘sufficient’.
Response: The word enough has been replaced with sufficient……Line 274

Lines 370-372: Please improve the syntax.
Response: The sentence construction of the whole discussion section has been revised.

Line 398: replace ‘condition’ with ‘comorbidity’ in keeping with the context of the paragraph.
Response: The sentence construction of the whole discussion section has been revised.

Line 402-406: Please explain why a longer duration of diabetes would be associated with DR screening uptake. Simply reporting data from other studies does not contribute to a quality Discussion.
Response: the following statements have been added to improve the quality of the discussion…. Patients that have a longer history of DM (particularly of at least 10 years) have a higher risk of developing sight-threatening DR [28]. Thus it may be expected that they are more likely to be referred for DR screening or to have a higher perceived need for undergoing DR screening. (Line 284 to line 287)

Line 407: Please include the reference for other low-income countries.
Response: The sentence construction of the discussion section has been revised. All sections stating low income countries has been referenced (line 216 to line 217, line 245 to line 247)

Lines 409-411: How does this impact/contribute to DR screening uptake? And Line 420: ‘Commitment’ in terms of what? (Resources, funding, skills, etc.) Please expand briefly.
Response: We had incorporated this paragraph into the discussion based on Reviewers’ advice to comment on the scalability of the DR screening program in Malawi. We found it hard to understand the advice and we felt that it was beyond the scope of our work but we still tried to discuss the scalability of the program. However, considering the difficulty with synchronizing the concept of scalability to the theme of our discussion, we would like to request that we be allowed to remove this paragraph as we had done  in our first draft.
Please be consistent with your spelling. I suggest using UK English uniformly e.g., ‘programme’, ‘glycaemic’, etc.
Response: The manuscript has been updated to UK English version
Line 424: improve the grammar; this is confusing.
Response: The sentence construction of the whole discussion section has been revised.
Line 425: Replace ‘studied’ with ‘established’.
Response: studied has been replaced with established (line 301)

Line 429: Reports from which countries? Does it include Malawi?
Response:The line has been revised to state the countries and reads as follows;  Previous reports from Kenya, Nigeria and mostly high in come countries have shown that increasing age and low visual acuity are associated with increased attendance for DR screening [13,19,32,33] (line 305 to line 307)
Line 447: ‘secondary level’ what?
Response: screening at secondary level health facility…. Line 384

Lines 457-458: Is this feasible/ practical in Malawi where there is likely a shortage of skilled staff who are overburdened with a large volume of patients? The current trend is toward task-shifting i.e., to lower-level health personnel; however, the authors are now suggesting the reverse.
We have changed the reference and edited the statement to clarify the point that we are proposing for primary healthcare workers to be the cadre of personnel offering education for DR whilst providing care for DM (Line 261 to line 263). We do acknowledge that this task-shifting towards personnel offering DM care may increase their workload. Thus we call for research into the feasibility of integrating DR education into DM care with the aim of finding the method that is effective for the rural setting.
